# "The non-ischemic repair" as a safe alternative method for repair of anterior post-infarction VSD

**DOI:** 10.1186/1749-8090-5-6

**Published:** 2010-02-19

**Authors:** Efstratios E Apostolakis, Antonios Kallikourdis, Nikolaos G Baikoussis, Panagiotis Dedeilias, Dimitrios Dougenis

**Affiliations:** 1 Cardiothoracic Surgery Department. Patras University School of Medicine, 26500 Rion Patras, Greece; 2 Aberdeen Royal Infirmary, Aberdeen, UK; 3 1st Cardiac Surgery Department. "Evangelismos" General Hospital of Athens, Athens, Greece

## Abstract

Patient's myocardium with post-infarction ventricular septum defect (VSD) is characterized by severe dysfunction. The "additive ischemia" caused by the operating process of cross-clamp ischemia and reperfusion injury, has a significant aggravation to the myocardium and overall negative impact to patient's outcome. We present a useful, safe and advantageous methodology in order to abolish "the toxic phase" of ischemia-reperfusion which is adopted by most as the "classic repair method" of myocardial protection. This abolition is in our opinion, particularly beneficial in order to reverse postoperatively the Low Cardiac Output Syndrome (LOS) and achieve better short and long term results. By using this method we avoid the aortic occlusion, the use of systematic hypothermia and any cardioplegic arrest. Furthermore, the total cardio-pulmonary bypass (CPB) time is significantly reduced, tissue debridement and stitching is much easier and safer. We think the method is applicable for every anterior and apical case of post-infarction septum rupture. After application of method in 3 patients with anterior post-myocardial infarction VSD, we are convinced that the patient will have a better postoperative haemodynamic condition and therefore a better outcome.

## Introduction

The rupture of the interventricular septum after myocardial infarction constitutes a severe mechanical complication of the coronary artery disease with very high surgical mortality (19-50%) and morbidity [1,2]. Many factors contribute to an unfavourable surgical outcome such as the emergency, the coexisting 3-vessel coronary artery disease, the posterior rupture, the "non-complete revascularization" operation, the "intractable" shock and the secondary organ-failure (mainly renal) [2,3]. The adequate myocardial protection during the operation is considered to be the cornerstone for a better outcome postoperatively [4,5]. The classic method of systemic hypothermia, aortic occlusion, and intermittent administration of cold blood cardioplegic solution is a well established method for the reconstruction of the post-infarction VSD [1-4,6]. Nevertheless, cardioplegic arrest is related to perioperative myocardial injury, which is considered as a severe determinant of postoperative haemodynamic condition, and therefore of clinical outcome [7,8]. This is the reason of suggestions by some authors for other alternative methods as that of using continuous myocardial perfusion after aortic occlusion, or by using intermittent ventricular fibrillation, or by administration of normothermic cardioplegia [9,10]. We propose another alternative method of myocardial protection during surgical repair of the anterior or apical cases of ruptures of the ventricular septum, and we recommend this as simple, safe and efficient.

## Technical Aspects

**Figure 1:**
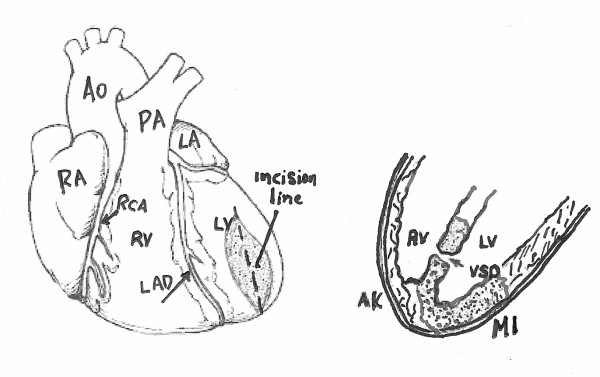
**After the left ventricle evacuation, we inspect the left ventricle wall to identify her thinner portion in order to perform the proper incision-ventriculotomy 3-4 cm of length**.

**Figure 2:**
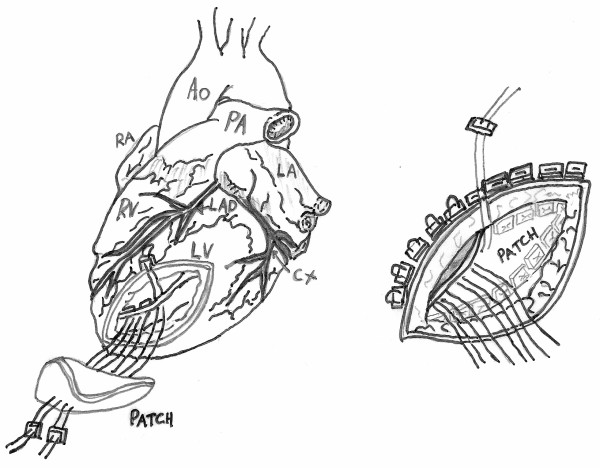
**At the end of the necrotic tissue remotion, the rupture is circumferentially repaired by using intermittent 3-0 Prolene sutures reinforced with pledgets from the site of the left ventricle, through a Dacrom patch up to the epicardium in "U shape" fashion**.

**Figure 3:**
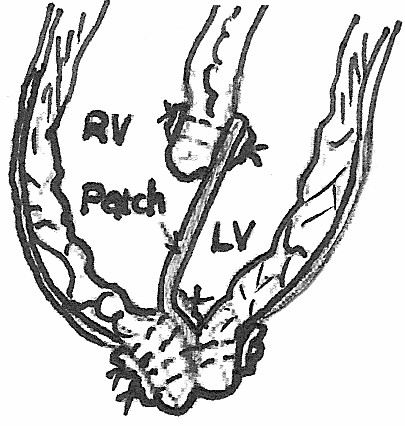
**Soon after the deairing of left cardiac chambers, we tight down all the sutures and securely close the ventriculotomy**.

The sternotomy is carried out slightly leftwards of the middle line for better approach of the left ventricle and of the apex. We use deeper stay sutures "to hold" the pericardium higher in order to have better exposure of the cardiac cavities. These steps will significantly help the following left ventricular manipulations although sometimes they restrict in a certain degree the handling of the right atrium. For the patient's connection to the CPB circuit we use the classic ascending aorta cannulation and a typical bicaval cannulation through the right atrium. No other catheter is required, a fact that facilitates further more the following surgical manipulations. Systematic hypothermia is not applied and the operation is carried out on normothermia. The patient is in Trendelenburg's position when we commence the CPB. Right after full flow on CPB we expose the left ventricular apex and through a small incision we insert a left ventricular vent. The left ventricular venting decompresses the left ventricle as well as the lungs. After the left ventricle evacuation we inspect the left ventricle wall to identify her thinner portion in order to perform the proper ventriculotomy (figure 1). The initial length of the ventriculotomy is 3-4 cm but it can be extended furthermore, as required after the inspection of the septum and the left ventricular cavity from inside. We routinely place surgical gauze beneath the heart in order to appropriately elevate the apex and expose the site of rupture, as much as needed to avoid possible distortion of aortic valve and sequent regurgitation. The latter will be evident by the back-flow of blood through the outflow-tract of left ventricle. The inspection concerns the position of the rupture, its margins, and the viability of the surrounding anatomical structures such as the anterolateral papillary muscle, the lateral ventricular wall, the inferior ventricular wall, etc. Gradually we remove most of the necrotic tissues around the edges of the rupture up to the point where the first bleeding from the viable myocardium (bleeding tissues) will appear. The rupture is then circumferentially repaired by using intermittent 3-0 Prolene sutures reinforced with pledgets from the site of the left ventricle, through a Dacron patch up to the epicardium in "U- shape" fashion (figure 2). After completion of stitches and before tight them, a second vent is inserted through the right superior pulmonary vein and under direct vision, it is properly placed in the left ventricle. Then, the anesthetist repeatedly inflates the lungs (Valsalva maneuver) till the left ventricle be filled by blood in order to de-air the left cardiac chambers. Then we tight down all the sutures and securely close the ventriculotomy (figure 3). The second vent starts functioning and the left ventricle is left to beat empty of volume. The last part of the operation is carried out using an off pump coronary artery bypass (OPCAB) stabilizer in order to perform the necessary distal coronary anastomoses and subsequently the proximal by partial clamping of the aorta (for the cases with more than one graft). After completion of the proximal anastomoses the extracorporeal circulation is interrupted and hemostasis is performed according to the standard method.

## Discussion

Patient's myocardium with post-infarction VSD is characterized by severe dysfunction [2,3]. Many unfavourable factors such as the recent infarction, the shock condition, the increased tissue (myocardial) edema, the inotropic support, the increased endogenous produced catecholamines, as well as the coexisting hypoxia due to pulmonary congestion are causing severe malfunction of the rest "rescued" myocardium. The additional ischemia to this myocardium, due to aortic occlusion and systemic and local hypothermia, entails significant postoperative functional deterioration and finally, possible unfavourable outcome. The methodology of myocardial protection using obligatory aortic occlusion, continuous or even intermittent, which was applied from the beginning of the surgical treatment of the post myocardial infarction mechanical complications, is still consider to be by many authors "inevitable" [1-3,5,6]. Even Gummert et al [6] in their chapter about the use of beating heart methodology in patients with acute myocardial infarction, state: "ventricular septal defect, acute mitral regurgitation, and myocardial free wall rupture following acute myocardial infarction require reparative surgery **under cardioplegic arrest**, and therefore will not be discussed any further in this chapter". The attempt to avoid systematic hypothermia, aortic occlusion and cardioplegia infusion is aiming to avoid cardiac arrest and to nullify the ischemic time. Our methodology has a series of significant advantages, especially important in our opinion for the early and also the late postoperative results: **a) **it does not aggravate the myocardium with the "toxic influence" of the ischemia - reperfusion process, **b) **additionally it does not have the adverse effect of the systemic hypothermia, **c) **it allows to the left ventricle to contract empty of volume on extracorporeal circulation, condition which consider to be the most favorable from the energy consumption point of view ("... the oxygen consumption of the beating, empty heart -as on cardiopulmonary bypass- is less than under any other condition.") [11], **d) **it significantly reduces the CPB time, another important detrimental factor, mainly because it avoids the hypothermia but also because we don't use any other catheter for cardioplegia infusion etc., **e) **it precludes possible complications from the cardioplegic infusion such as injury to the coronary vessels, coronary embolism, myocardial oedema etc., **f) **it allows easier distinction of the excision borders of the non-viable septum up to the point of the viable bleeding tissue, **g) **it secures safer "palpable feeling" for the proper setting and above all correct riveting of the sutures in a contracting not arrested myocardium which keeps the natural muscular tone (it avoids crushing the arrested myocardium), **h) **it can be applied in the anterior and apical ruptures which are the majority of the ruptures representing 60-80% of all cases [3], and finally **ι**) it allows seasonably control and correction of any local bleeding point in the ventriculotomy suture line during the phase of the passive lung expansion, and the temporary left ventricle overloading. Our method's disadvantage is that it can not be applied in the cases of inferior septal ruptures, unless they are either small or chronic, and the temporarily produced aortic regurgitation can be well tolerated by the patient. We have to note that there is no risk of aortic embolism during the maneuvers, because the existence of continuously positive intra-aortic pressure and patient's Trendelenburg position. Up today we have used the method in 3 patients with anterior rupture ascertaining the previous mentioned advantages in emergent setting. We observed a better global cardiac function during the early postoperative phase. It has been observed an amelioration of about 10% of the left ventricle ejection fraction. Two of the patients survived without complications and discharged after 13 and 17 days respectively from hospital, but unfortunately, the third one died 28 days postoperatively in intensive care unit (ICU) from multiple organ failure (MOF). The small number of our patients does not allow us to randomly compare the haemodynamic and clinical results, but we greatly believe that the complete abolition of the ischemic-time improves the safety conditions of the operation, the early results, as well as the survival in these patients. However, further multicenter randomized trials are necessary in order to establish the superiority of this method.

## Competing interests

The authors declare that they have no competing interests.

## Authors' contributions

EA performed the interventions, design the manuscript and revised it, AK designed the figures and corrected the manuscript, NB structured the manuscript and submitted it, PD participated in the interventions, DD participated in its design and coordination and all authors read and approved the final manuscript.
